# Advancements and Challenges in Eliminating Perinatal Transmission of HIV in Sub‐Saharan Africa: A Comprehensive Analysis

**DOI:** 10.1155/arat/8894456

**Published:** 2025-11-30

**Authors:** George Baah, Stephen T. Odonkor

**Affiliations:** ^1^ Ghana Institute of Management and Public Administration, Accra, Ghana, gimpa.edu.gh

**Keywords:** adherence, determinants of adherence, drug resistance, interventions, psychosocial support, virologic failure

## Abstract

UNAIDS estimated that in 2023, 84% of pregnant women living with HIV were receiving antiretroviral therapy (ART) for the Prevention of Perinatal Transmission of HIV (PPT‐HIV), potentially averting up to 220,000 new HIV infections in newborns. However, despite the scale‐up of PPT‐HIV services, approximately 160,000 children became infected with HIV in 2022 alone. This underscores the urgent need to refocus efforts on addressing the challenges of PPT‐HIV to ensure that sub‐Saharan Africa meets global targets for eliminating new perinatal transmission. This article examines interventions that have demonstrated positive impacts on PPT‐HIV services and recommends their implementation in areas where they are needed. Among these interventions is the critical importance of enhancing community‐based test‐and‐treat initiatives for people of childbearing age and improving access to prenatal care, HIV testing and ART for expectant mothers. Additionally, there is a need to emphasise the role of technology and involve male partners in PPT‐HIV programmes to enhance effectiveness and achieve the desired outcomes.

## 1. Introduction

Perinatal transmission of HIV (PT‐HIV) occurs when an HIV‐infected mother passes the virus to her child during pregnancy, childbirth or breastfeeding. In sub‐Saharan Africa (SSA), which bears a disproportionate burden of the global HIV epidemic, PT‐HIV remains a significant public health challenge [1]. Prevention of Perinatal Transmission also known Prevention of Parent to Child Transmission of HIV (PPT‐HIV) programmes aim to reduce transmission rates by offering HIV testing and counselling to pregnant persons, providing antiretroviral therapy (ART) to HIV‐positive people, and promoting safe infant feeding practices (such as exclusive breastfeeding for the first 6 months followed by safe complementary feeding until the child is 1 year old).

Without appropriate interventions, HIV‐positive pregnant people in Africa face a 15%–45% risk of transmitting the virus to their children during pregnancy, labour, delivery or breastfeeding [2]. However, advancements in ART have significantly decreased perinatal transmission rates globally. Pregnant persons living with HIV deserve person‐centred care to address their unique concerns and fears, aiming for normal pregnancies [3]. Early detection of HIV infection must be followed promptly by linkage to lifelong treatment and care, including support to remain in care while achieving viral suppression, as well as partner services. Eliminating PT‐HIV in SSA requires a multifaceted approach involving improved access to healthcare services, expanded ART coverage, overcoming barriers to care and fostering community engagement.

Global commitments and the integration of HIV prevention strategies into maternal, newborn, child and adolescent health services, along with strengthened health systems, have significantly advanced the goal of eliminating PT‐HIV. The UNAIDS estimated that since 2000, PPT‐HIV efforts have averted an estimated 3.4 million infections [4]. Countries such as Uganda have seen a dramatic decline in perinatal transmission rates, from 30% 2 decades ago to about 3% today, with the overall transmission rate at 18 months postpartum being 2.8%, below the national goal of 5% [5, 6]. Similarly, Botswana, a previously high disease–burdened country, has seen a dramatic decline in perinatal transmissions, achieving an HIV transmission rate under 5% and an HIV case rate of less than 500 per 100,000 live births [7]. According to UNAIDS data, for the first time since the start of the HIV pandemic, more new infections occurred outside of SSA than within it [8]. This progress is further supported by access to sexual and reproductive healthcare, prevention of unplanned pregnancies and screening and treatment of sexually transmitted infections among HIV‐positive women and girls [2, 9].

Every child born with HIV represents a failure in prevention. Despite the existence of effective treatment, challenges faced by pregnant and breastfeeding people living with HIV have impeded efforts to eliminate PT‐HIV. This review article aims to comprehensively analyse advancements and challenges in PT‐HIV elimination in SSA, informing policy and practice towards the ultimate goal of eliminating PT‐HIV in the subregion. The subsequent sections of this review article will delve deeper into these issues.

Although recent studies continue to advance the understanding of PPT‐HIV strategies, it is essential to acknowledge foundational reviews that shaped early policy and clinical guidance. Notably, De Cock et al. [10] provided a critical synthesis of PT‐HIV risks and interventions in low‐resource contexts, emphasising the translational impact of research on public health practice [10]. More recently, Teasdale et al. [1] offered multicountry evidence on retention in care and viral suppression among pregnant and breastfeeding women, further reinforcing the importance of sustained engagement in HIV services. This review builds upon this foundation by examining more recent programmatic outcomes, highlighting persistent gaps in retention and viral suppression among pregnant and breastfeeding persons despite broader ART coverage. By contextualising emerging data within the continuum of evidence from earlier and contemporary studies, this review aims to identify persistent barriers and inform adaptive strategies to enhance PPT‐HIV programme effectiveness in SSA.

## 2. Overview of the HIV Epidemic in SSA

In 2023, an estimated 65% of the 39.9 million PLHIVs globally were from SSA, which accounted for 640,000 of the 1.3 million new infections and 380,000 of the 630,000 AIDS‐related deaths reported [8]. Women and girls accounted for 44% of all new infections in 2023 globally, with adolescent girls and young women in SSA being more than three times as likely to acquire HIV as their male peers [8]. In SSA, women and girls of all ages represented 62% of all new HIV infections, whereas more than 73% of new HIV infections in 2023 were among men and boys in other parts of the world [8].

In 2023, an estimated 67% of the 38.4 million PLHIVs globally were from SSA, which accounted for 660,000 of the 1.3 million new infections and 380,000 of the 630,000 AIDS‐related deaths reported [11]. Women and girls accounted for 46% of all new infections in 2022 globally, with adolescent girls and young women in SSA being more than three times as likely to acquire HIV as their male peers [11, 12]. Adolescent girls and young women accounted for more than 77% of new infections among young people aged between 15 and 24 years [4, 11]. According to UNAIDS [4, 11]), in 2022, 4000 teenage girls and young women between the ages of 15 and 24 worldwide contracted HIV each week, with SSA accounting for about 3100 of these. The drivers of transmission include low condom use, high drug use and stigma [13]. Despite progress in reducing new infections and AIDS‐related mortalities since the peak in 2004, HIV still imposes a significant health burden on countries in SSA [4, 11]. Compared to other locations, SSA has a far greater adult prevalence for HIV/AIDS, with rates ranging from less than 0.1% in countries such as Somalia, Comoros and Sudan to as high as 17.8% in South Africa, 19.3% in Lesotho and 25.9% in Eswatini [14]. In recent years, HIV prevalence has decreased in countries such as Uganda, while it has been high in others, such as Nigeria [14]. The effects of the epidemic differ throughout countries and regions, necessitating specialised treatment and prevention initiatives to handle the range of issues raised by HIV/AIDS in SSA [15, 16].

### 2.1. Importance of PPT‐HIV IN SSA

PT‐HIV in SSA remains a significant concern that contributes to the region’s high burden of HIV/AIDS. Only SSA accounts for more than 65% of the global burden of HIV infection, with women disproportionately affected and representing 62% of PLHIVs [17–19]. The primary mode of HIV transmission in this region is through heterosexual sex, leading to a concomitant epidemic in children through vertical transmission. Therefore, the dire burden of HIV could be altered if PT‐HIV is successfully eliminated in the region and globally making it an area for intervention [17].

The UNAIDS estimated in 2023 that 84% of pregnant women living with HIV were receiving ART, which was expected to avert an estimated 220,000 newborn HIV infections (UNAIDS, 2024a). Despite these expected gains from the PPT‐HIV programme, an estimated 120,000 [83,000–170,000] children were newly infected with HIV in 2023 alone [18]. This, therefore, demands renewed focus on preventing vertical transmission in mothers and newborns. Even though there has been progress in PPT‐HIV, challenges still persist in meeting global targets for eliminating new PT‐HIV.

It is worth noting that PPT‐HIV has led to the reduction of the incidence of annual AIDS‐related deaths among children by 75% from its peak in 2003 [16]. In countries such as South Africa, the PPT‐HIV programme aims to reduce HIV prevalence among pregnant women and eliminate PT‐HIV, which has led to a reduced intrauterine transmission to under 1% by 2013 and maintain low levels thereafter [20, 21]. However, other African countries continue to record high incidence of PT‐HIV rates, such as Ethiopia (8.1%) and Nigeria (2.74%) [22]. To combat PT‐HIV effectively, there is an urgent need to improve community test‐and‐treat campaigns among people of reproductive age. Enhancing access to antenatal care, HIV testing and ART for pregnant people is crucial for reducing vertical transmission of HIV and achieving the goal of eliminating PT‐HIV [22].

### 2.2. Historical Context of PT‐HIV Elimination Efforts in SSA

It is necessary to understand the historical context of PT‐HIV in SSA to appreciate the progress and challenges faced in PT‐HIV elimination efforts in SSA. The first cases of PT‐HIV were reported in the 1980s, raising concerns about the impact of HIV on children born to infected mothers. There was minimal knowledge and interventions at the time; therefore, understanding effective PT‐HIV prevention strategies was restricted. Initial therapies, starting in 1994, such as zidovudine (AZT) monotherapy during pregnancy, could reduce the risk for vertical transmission by 67% but were complicated and had adverse effects [23]. In the early 2000s, the global focus was towards expanding PPT‐HIV programmes due to the availability of effective ART regimens and increased awareness of PT‐HIV. The UNAIDS, WHO, UNICEF, and UNFPA launched the “PPT‐HIV programme in 2001 and then followed by the “3 by 5” initiative in 2004. This programme aimed to provide ART to three million people in low‐ and middle‐income countries by 2005. This was meant to significantly impact pregnant women and reduce PT‐HIV [24, 25]. Challenges such as poverty, stigma and limited access to healthcare continued to exist even though PT‐HIV rates were reduced in the 2010s. To accelerate efforts to eliminate perinatal transmission by 2020 altogether, the “Eliminating Perinatal Transmission Global Alliance” was established in 2016. Notwithstanding the progress made over the years, PT‐HIV persists [26, 27]. There is, therefore, the need for ongoing efforts to improve healthcare access, remove social barriers and conduct research to advance prevention and treatment in the future.

## 3. Advancements in PT‐HIV Elimination—ART and its Role in Reducing PT‐HIV

ART plays a crucial role in reducing PT‐HIV in SSA. Early effective preconception and antenatal ART reduce intrauterine and intrapartum PT‐HIV, while maternal postpartum ART reduces postnatal PT‐HIV [28]. In Namibia, the introduction of lifelong ART for PPT‐HIV led to a significant reduction in PT‐HIV, with the estimated number of incident infant HIV infections declining from 712 in 2010 to 201 in 2014 [29]. In Rwanda, the adoption of ART for all pregnant and breastfeeding women in 2010 and the extension of ART for all pregnant women beyond the breastfeeding period to lifelong therapy in 2012 contributed to a low PT‐HIV rate of 1.58% [30]. According to the WHO, as of 2023, approximately 84% of pregnant women and girls living with HIV worldwide had access to ART for PPT‐HIV [31]. However, problems such as late HIV diagnosis, poor adherence to ART and disruptions caused by health emergencies such as the COVID‐19 pandemic have hindered the efficiency of PPT‐HIV programmes in some regions [30, 32].

### 3.1. The Expansion of PPT‐HIV in SSA

In SSA, PPT‐HIV programmes are essential for lowering the risk of HIV transmission from infected parents to their offspring. These programmes offer many advantages, such as helping women maintain their health, preventing unintended pregnancies and protecting their unborn children from HIV infection [33]. Between 2010 and 2018, PPT‐HIV programmes helped to avoid about 1.4 million HIV infections among children around the world. ART drastically reduces maternal HIV transmission to newborns to less than 5%, compared to a 15%–45% transmission risk without ART [33].

Notwithstanding the progress and the documented benefits of PPT‐HIV, challenges exist in the uptake of these services in SSA. For instance, ART coverage for pregnant women living with HIV remains below 50% in countries such as Angola and between 50% and 69% in others, such as Ghana, Ethiopia, and the Democratic Republic of Congo [33]. However, according to studies conducted in several states in South Africa before the COVID‐19 pandemic, HIV testing uptake was as high as 99.8%, and more than 95% of HIV‐positive pregnant women were on ART [22, 34]. The research in SSA suggests that women’s awareness is limited despite the proven evidence that knowledge leads to better outcomes in PPT‐HIV [35]. A Tanzanian study found that just 46% of mothers understood PT‐HIV and PPT‐HIV adequately, while studies in Ghana, Ethiopia and Cameroon found that only 10%, 22.4% and 23.7% of women knew about PPT‐HIV [36–39]. Efforts to increase PPT‐HIV knowledge and coverage are essential. Empowering women through decision‐making power has positively influenced PPT‐HIV knowledge and uptake [22]. Strengthening policies and interventions to enhance people’s access to information and services, improving ART coverage for pregnant people living with HIV and addressing barriers to PPT‐HIV uptake are crucial steps towards further reducing PT‐HIV.

### 3.2. Impact of PPT‐HIV Programmes on PT‐HIV Rates in the Region

The expansion of PPT‐HIV programmes has significantly reduced the rate of new HIV infections among children by approximately 62% from 2010 to 2023, and specifically, new HIV infections in children under five years from around 300,000 in 2010 to about 120,000 in 2023 (UNAIDS, 2024a; [27]). Astawesegn et al. [26] reported that the PT‐HIV rate in SSA decreased by 38% from 27.18% in 2010 to 16.90% in 2019, representing a decline of 1.15% per study year in the region. With the implementation of PPT‐HIV programmes, the eligibility requirements for starting ART were improved, increasing coverage with estimates around 93.7%. For example, a study in Zambia found ART coverage among pregnant women living with HIV to be 93.7% between 2016 and 2019, with 97.7% of women diagnosed before pregnancy and 88.2% of those diagnosed during pregnancy receiving treatment during pregnancy[40]. PPT‐HIV interventions have achieved significant reductions in paediatric HIV infections across several African countries, including 71% in Namibia, 76% in Malawi, 85% in Botswana, 83% in Rwanda, 69% in Zimbabwe and 65% in Uganda, while also reducing AIDS‐related child deaths by approximately 43%–62% since 2010 in SSA nations ([41]; UNAIDS, 2024a; [42]).

The 2021 Global AIDS Update by UNAIDS reported that Eastern and Southern Africa have achieved the most significant progress in reducing PT‐HIV rates, with a 73% decline between 2010 and 2020 [43]. The expansion of PPT‐HIV services has contributed enormously to a progressive reduction in new paediatric HIV infections [44]. Nonetheless, there are significant differences in PT‐HIV rates between countries, with some having PT‐HIV rates of less than 5% while others have PT‐HIV rates of more than 30%. ART coverage during pregnancy is a significant predictor of the rate of HIV transmission from mother to child, with countries that have higher ART coverage typically having lower rates of perinatal transmission [26].

### 3.3. New Technologies and Their Potential to Improve PT‐HIV Elimination

Emerging technologies present unprecedented opportunities to accelerate the elimination of PT‐HIV through innovative interventions that address gaps in the current prevention strategies. These cutting‐edge approaches include point‐of‐care viral load testing and rapid drug resistance assays that enable real‐time monitoring and treatment optimisation. Others include digital health platforms that facilitate remote patient monitoring, novel therapeutic formulations including long‐acting antiretroviral drugs that reduce dosing frequency and improve compliance and artificial intelligence–driven predictive models that identify high‐risk populations and optimise resource allocation.

#### 3.3.1. Technology‐Delivered Intervention Strategies

Digital tools, including social media, research websites, live chat, text messaging, mobile applications, online videos, electronic health records, vending machines, conversational agents, virtual reality and the possible use of blockchain technology and mobile payment services, are employed in these strategies to increase the number of HIV tests [45].

#### 3.3.2. HIV Self‐Testing (HIVST) With Digital Support

This strategy links key and general populations to care by increasing HIVST uptake, participant preference and connection to care through social media and app‐based interventions [46]. In both critical and general populations, HIVST with digital support strategies showed success in terms of uptake, participant preference and care access. Digital innovations were well‐received in various contexts and were especially effective in addressing hard‐to‐reach communities and first‐time testers. Although the social media platforms that are now available to promote HIVST showed promise, further research on HIVST and improvements in digital assistance among specific populations, especially in pregnant people, is required [46]. This has improved test uptake and linkage to care among men and key populations who often face barriers with traditional facility‐based testing. Acceptability has been generally high, ranging from 22% to 95%, with men, female sex workers and young adults showing strong preference due to reduced stigma, privacy and convenience [47–50]. According to Kra et al. [51] and Hlongwa et al. [50], HIVST has proven effective in engaging men, who often underutilise conventional testing services, and has increased maternal retesting rates by 35% among pregnant and postpartum persons and their partners. Although linkage to confirmatory testing after a reactive HIVST result varies, the outcomes are encouraging, with up to 95% of those confirmed HIV‐positive–initiating ART, and 91% seeking confirmatory testing within 3 months.

#### 3.3.3. WHO Toolkit for Quality HIV Testing Services

This toolkit supports countries in transitioning to new HIV testing algorithms, including the use of dual HIV/syphilis rapid diagnostic tests (RDTs) and the phasing out of western blotting methods [7]. Many countries have since adopted this WHO recommendation. For instance, HIV testing programme compliance increased in the WHO African region from 8% in 2014 to 71% in 2021. But some of the vacuums are still there. Western blotting processes are used in over a dozen nations, which hinder quick access to ART and PrEP as well as same‐day diagnosis [7].

#### 3.3.4. Advances in HIV Testing Technology

New laboratory‐based technologies are revolutionising HIV diagnosis, enabling more sensitive detection of HIV infection, especially during the acute phase when transmission risk is high. Some of these tests include microparticle enzyme immunoassay, a highly automated modern test based on ELISA technology, and microparticle EIA, which can generate results in less than an hour. These tests detect viral antigen‐antibody complexes and play a crucial role in early HIV diagnosis [52]. Other tests include polymerase chain reaction (PCR), which amplifies viral nucleic acids to detect HIV infection with high sensitivity and specificity. It is instrumental in identifying HIV in newborns born to infected mothers and plays a vital role in early diagnosis [52].

#### 3.3.5. HIV Vaccines

HIV vaccine remains another source of hope for future generations, with ongoing research showing some promise in curtailing the spread of the virus. One such research is the mRNA technology, inspired by the success of COVID‐19 mRNA vaccines. Other approaches include the development of mosaic vaccines, which combine components from multiple HIV strains to provide broader protection against the diverse nature of HIV [53, 54]. The mosaic vaccine, also known as HPX3002/HVTN 706, is a Phase 3 clinical trial that combines pieces of many HIV viruses to achieve a broader immune response. A study has found this experimental HIV vaccine regimen to be safe but ineffective in protecting against HIV acquisition [54, 55]. Despite this setback, the HIV research community remains committed to developing a safe and effective vaccine to end the HIV/AIDS pandemic.

## 4. Challenges in PT‐HIV Elimination

While significant progress has been made in reducing the PT‐HIV, several challenges still hinder the complete elimination of this issue, particularly in SSA, the region most heavily impacted by HIV. These challenges can be broadly categorised into health system barriers, sociocultural and gender barriers and maternal health and nutritional challenges.

### 4.1. Health System and Service Delivery Barriers

Access to ART and PPT‐HIV services remains uneven across SSA. Health system limitations, including infrastructure deficits, workforce shortages and service disruptions, especially during crises such as the COVID‐19 pandemic, have hindered efficient care delivery [56–58]. Limited awareness about HIV transmission and the importance of ART continues to contribute to suboptimal uptake of PPT‐HIV services. Furthermore, the lack of reliable data and monitoring systems impedes effective evaluation and adaptation of care programmes [58, 59].

Other barriers to care programmes include gaps in ART initiation and reinitiation among people of reproductive age. Many patients lack prior exposure to ART, which limits the effectiveness of PPT‐HIV efforts [60]. To improve outcomes, a comprehensive understanding of local service delivery contexts and continuous investment in infrastructure and workforce development are needed.

### 4.2. Sociocultural and Gender Barriers

Stigma and discrimination remain pervasive challenges in the fight against PT‐HIV. Fear of being judged, isolated or losing social status deters many people from seeking HIV testing, disclosing their status or adhering to ART [61, 62]. Stigma has a cascading effect across the PPT‐HIV cascade, from testing and disclosure to adherence and retention, ultimately increasing the risk of HIV transmission to infants [59, 63, 64]. Gender inequality and intimate partner violence further exacerbate these issues. Power imbalances, cultural norms that limit people’s autonomy and intimate partner violence reduce people’s ability to protect themselves from HIV and access care [63, 65, 66]. According to Leddy et al. [67] and Maman et al. [68], fear of violence or actual experiences of violence often deter women from disclosing their HIV status or remaining in treatment, which in turn has adverse consequences for both maternal and child health outcomes [67, 68]. Addressing these sociocultural and gender‐based barriers is critical to improving PPT‐HIV outcomes.

### 4.3. Maternal Health and Nutritional Challenges

Pregnant and breastfeeding women living with HIV often face complex nutritional and health challenges that impede their ability to adhere to PPT‐HIV protocols. Malnutrition and food insecurity compromise both maternal and infant health, increasing susceptibility to infections and undermining the effectiveness of ART [69–71]. Food‐insecure parents are more likely to miss doses or discontinue ART and less likely to breastfeed exclusively, increasing the risk of PT‐HIV.

Furthermore, poor maternal nutrition has been linked to mental health issues such as anxiety and depression, which further reduce engagement with health services [72]. Breastfeeding challenges, including lack of support systems and breast infections, complicate infant feeding practices. While WHO recommends exclusive breastfeeding for the first 6 months, followed by continued breastfeeding with complementary foods, adherence to this guidance is often compromised in resource‐limited settings, with studies such as the one conducted in Ethiopia reporting that 20% of HIV‐exposed neonates received mixed feeding [37, 73, 74].

### 4.4. Retention in Care and Continuity of Services

Low retention of mothers and infants in PPT‐HIV programmes remains a barrier to the elimination of PT‐HIV. Factors such as transportation costs, clinic wait times, stigma and fear of disclosure lead to early loss‐to‐follow‐up, particularly during the antenatal and postnatal periods [75, 76]. Health facility–level issues, including staffing shortages and long distances to clinics, further hinder continuity of care. Community‐based interventions, such as peer support groups, mobile health technologies and engagement of traditional leaders, have shown promise in improving retention and adherence [77–79]. Tailored, scalable strategies including text message reminders, conditional cash transfers, male partner involvement and mentor‐mother support are needed to ensure continuous care and viral suppression [80]. By addressing these multifaceted challenges through integrated approaches, stakeholders can enhance the effectiveness of PPT‐HIV programmes and move closer to the global goal of eliminating PT‐HIV.

## 5. Strategies to Overcome Challenges

Despite the enormous progress made in the PPT‐HIV, there are still many obstacles to overcome. These obstacles include social stigma, gender inequity, dietary inadequacies and health inequities, all of which affect people’s access to and compliance with preventive measures. We shall discuss creative approaches to some obstacles to hasten the global objective of PT‐HIV.

### 5.1. Strengthening Health Systems and Improving Access to ART and PPT‐HIV Services

Strengthening health systems and improving the delivery of HIV prevention services are foundational to eliminating PT‐HIV. In many SSA settings, healthcare systems are under‐resourced, with deficits in infrastructure, ART supply chains and skilled personnel, especially in rural areas [81]. Targeted investments are required to expand healthcare infrastructure, decentralise ART and PPT‐HIV services and increase the availability of trained health professionals through task‐shifting and continuous capacity building [82, 83].

The decentralisation of ART services to primary care facilities has improved access in countries such as Malawi, Uganda and Zimbabwe [84]. Strengthening health information systems for real‐time monitoring of maternal and infant outcomes is another way to enhance programme efficiency and accountability [85]. Community outreach, culturally sensitive health education and integration of HIV services with maternal and child health programmes are also required for increasing service uptake.

Electronic medical records (EMRs), point‐of‐care diagnostics and mHealth solutions (e.g., SMS reminders and digital adherence tools) can be adopted to streamline service delivery, enhance data quality and improve maternal engagement [86, 87].

### 5.2. Improving Retention in PPT‐HIV Programmes Through Peer Support and Mentorship

Sociocultural norms, stigma and gender inequality are major barriers to the success of PT‐HIV programmes. Women may face violence or abandonment upon HIV disclosure, limiting their willingness to seek care or adhere to treatment [68]. Addressing these challenges requires gender‐sensitive programming, including providing care in nonjudgemental environments with trained female healthcare workers, ensuring confidentiality and creating safe spaces for disclosure and treatment [88].

Empowering women through education, vocational training and financial independence enhances their autonomy in health decision‐making [33, 89]. At the community level, behaviour change communication and engagement of traditional and religious leaders can challenge stigma and foster supportive environments for people living with HIV.

Engaging men in PT‐HIV efforts is equally important. Male involvement has been shown to improve maternal adherence and reduce stigma. Strategies such as weekend clinics for working men, couple counselling and male‐friendly health education campaigns can promote shared responsibility in HIV prevention [90, 91].

Legal protections and access to justice will enable people living with HIV to seek and continue care without fear of violence, discrimination or stigma. Strengthening legal frameworks against gender‐based violence and discrimination helps create safer environments where people can access HIV services confidently [4, 92, 94].

### 5.3. Addressing Health and Nutritional Challenges Through Integrated Health Services and Nutrition Support

Maternal malnutrition and poor health status undermine the effectiveness of ART and increase the risk of poor maternal and neonatal outcomes. In many resource‐limited settings, people face chronic food insecurity, micronutrient deficiencies and frequent illness—all of which compromise treatment outcomes [69, 95]

Integrated service delivery interventions are required to address these gaps by embedding nutritional support such as supplementation, counselling and food aid within HIV care and antenatal services, with special emphasis on micronutrient supplementation (e.g., folate, iron and vitamin A) for HIV‐positive pregnant people due to their heightened metabolic needs and vulnerability to infections.

Health education and counselling must be tailored to address the specific dietary needs of PLHIVs. Community‐based nutrition support, including food vouchers or agricultural initiatives, can also improve food security for HIV‐affected households.

There is also a need to strengthen maternal health services, including skilled birth attendance, prevention and management of coinfections (e.g., malaria and TB), and postpartum care, which will ensure holistic support for people and their infants within the HIV continuum of care.

## 6. Conclusion and Recommendation

Parent‐to‐child transmission of HIV continues to drive the growth of HIV infections in SSA. It accounts for around 11.2%, with perinatal transmission at 6.2% and postnatal transmission at 5.0%. Despite the progress made, the SSA response did not meet the global target of reducing perinatal transmission to less than 15,000 children even though there has been a significant 56% decrease since 2010, driven by gains in the use of effective ARVs before, during and after pregnancy.

Advancements in technology such as HIVST, which are connected to mobile technology, the use of EMRs, communication through mobile applications and text messages and enhanced databases for PPT‐HIV services, have been employed to advance the course of eliminating PT‐HIV. These tools are beneficial in countries where they have been used and could be adopted by other SSA countries to support their PPT‐HIV programmes.

The use of technologies and the implementation of enhanced PPT‐HIV services alone may not ensure that SSA achieves the target of elimination of PT‐HIV by 2030. Other challenges, such as gender inequity and intimate partner violence, HIV‐related stigma and sociocultural practices, infrastructural challenges, food insecurity and poverty, need to be addressed. These, in most cases, are the drivers of HIV infections and barriers to adherence to effective utilisation of PPT‐HIV services.

Policymakers need to develop and implement policies to ensure access to ART for pregnant people to prevent vertical transmission of HIV and allocate resources and funding for primary HIV prevention, treatment and care programmes. They must also monitor and evaluate the progress made in reducing PT‐HIV rates and promptly address any challenges that arise [44, 96].

Stakeholders must work with policymakers and healthcare practitioners to increase financing and support for HIV prevention, treatment and care programmes. They must implement and scale up initiatives that guarantee that pregnant people start and remain on ART even after birth. Additionally, efforts must be made to combat drug resistance by monitoring and analysing the prevalence of HIV drug resistance (HIVDR). There is a need for collaboration with healthcare providers and community leaders to promote awareness about the importance of ART adherence and PPT‐HIV.

Implementing these strategies is essential for achieving the WHO 2030 targets for the elimination of perinatal HIV transmission. By ensuring equitable access to ART, leveraging technological innovations, addressing structural barriers and fostering collaboration across healthcare systems and communities, SSA can accelerate progress towards reducing new paediatric HIV infections to near zero.

## Disclosure

All authors have agreed to publish this work.

## Conflicts of Interest

The authors declare no conflicts of interest.

## Funding

This research did not receive any specific grant from funding agencies in the public, commercial or non‐profit sectors.

## Data Availability

The data that support the findings of this study are available on request.
